# Progress in State Medicine

**Published:** 1896-10-17

**Authors:** 


### Progress in State Medicine.

The increased importance attached to the study of public health is strikingly demonstrated by the number of conferences which have been held during the past two months. The Sanitary Institute met at Newcastle, the British Institute of Public Health at Glasgow, the Scottish Sanitary Association at Dumfries, and the Public Health Section of the British Medical Association at Carlisle.

Professor Ramsay's address at Glasgow on "The Purification of the Atmosphere" (*Journal of State Medicine*, Sept., 1896) was replete with information and suggestion. Dealing with the smoke nuisance, he argued against the assumption that there is any real economy to be gained by the use of smoke-consuming grates or furnaces. The loss incurred by the escape of smoke is not, he said, more than 1 per cent. of the total heating effect, and it is on hygienic grounds alone that a remedy can be demanded. Smoke consisting, as it does, of finely divided particulate matter, determines, as the researches of Mr. Aitkin have demonstrated, the condensation of moisture. In this way sunlight is not only diluted by being compelled to pass through a pall of smoke, but may be entirely shut off by the resulting mist, fog, cloud, or rain. The rays which are most easily shut off are those of the blue and violet end of the spectrum, and these are precisely those which are fatal to microbial life, and which are engaged in the formation of hydrogen peroxide, the most potent destroyer of organic matter. Professor Ramsay would substitute coke for coal for both domestic and manufacturing purposes. In the home he suggested the use of a swinging gas bracket beneath the grate, by means of which the coke fire is easily ignited.

A recent important addition to our knowledge of atmospheric chemistry is supplied by the monograph of Drs. Billings, Weir-Mitchell, and Bergey, "The Composition of Expired Air and its Effects on Animal Life" (Washington: The Smithsonian Instit., 1895). These workers confirm to some extent the views of Haldane and Stewart, viz., that expired air contains no peculiar, poisonous, organic matter. Its ill effects are solely the result of a diminished proportion of oxygen, and an increased amount of carbon-dioxide, aided no doubt by the high temperature, and evil smells, the result of cutaneous exudation, which are always associated with "stuffy" rooms.

Dr. W. H. Corfield in his new work "Disease and Defective House Sanitation," gives it as the result of his experience that sore throat, diphtheria, and enteric fever are the diseases most commonly associated with defective drains and sanitary fittings. In this connection it is interesting to note that Dr. Newsholme ("Enteric Fever and Shellfish," *B. M. J.*, Sept. 12th, 1896) attempts to minimise the importance of the part played by drain effluvia, and regards infection as being for the most part due to the ingestion of contaminated food or drink. After carefully excluding the possibility of infection from milk, or from defective drainage, thirty per cent. of the cases of enteric fever in Brighton during the years 1893 to 1896 were attributable to shell-fish. Thirty-six cases were traced to oysters and thirty to mussels. The sufferers were chiefly males, adults, convalescents from acute diseases, and persons following such occupations as publican, caterer, theatrical manager, &c. Others having eaten oysters escaped enteric fever, but were attacked with diarrhœa and colic, excited by the presence of various coliform organisms. That such growths are often present in contaminated waters is confirmed by McWeeny (*B. M. J.*, Sept. 12th, 1896), who, investigating the water supply of Dublin Barracks, failed to find B. typhosus, yet demonstrated that the more highly nocuous water contained the greater proportion of coliform bacilli. The case against shell-fish is on the way to be established beyond all controversy, and the whole evidence will be found carefully analysed in Cartwright's report to the British Medical Association, now in course of publication. Oysters are laid down to fatten in estuaries and mouths of rivers where contamination is only too easy. It is now certain that the bacillus of enteric fever can preserve its vitality in fresh or sea water, and even in diluted sewage, for as long a period as six or eight weeks. The obvious remedy is to prevent the entrance of sewage into rivers and streams, but the powers of the Rivers Pollution Act are seldom enforced. In America this matter, and the treatment of sewage generally, are receiving much attention. Satterthwate ("How to Prevent River and Stream Pollution," Trans. Med. Soc. N.Y., 1896), after pointing out that already the crude sewage of New York and Brooklyn is causing serious nuisance to communities below, *e.g.*, Coney Island and Sheepshead's Bay, proceeds to discuss the various methods of sewage treatment. Irrigation, as practised in Paris and Berlin, is rejected as likely to break down, and as being a constant source of nuisance. The resulting effluent is satisfactory, but it is claimed that a system of precipitation and filtration, such as carried out at Glasgow, has both wider possibilities of application, and is cheaper. Milk of lime and sulphate of alumina are the substances used, and the effluent passes through successive filters of coke, sand, and gravel. The improvement in the condition of the Clyde since the above method was adopted is very marked. Ward ("Canals and their Impurities," *Sanitary Record*, Sept. 18th) draws attention to the condition of canal waters, a fruitful source of disease as witnessed by the Local Government Board Report on Disease in the Trent Valley.

Hill's (*Lancet*, Sept. 12th, 1896) account of diphtheria in Birmingham affords further evidence of our ignorance as to the causation of such epidemics. From 1873-94 the disease was quiescent. In 1895 it began to increase by leaps and bounds. No cause could be assigned. The smaller houses suffered more than the larger, and those with pan-closets more than those with water-closets but with these exceptions neither sanitary surroundings nor locality exerted any influence. Milk could be excluded as a cause, and also school attendance. Smith (*Sanitary Record*, Aug. 7th, 1896), indeed, holds that generally school attendance is an unimportant factor in the causation of diphtheria.

